# Using Lean Six Sigma techniques to improve efficiency in outpatient ophthalmology clinics

**DOI:** 10.1186/s12913-020-06034-3

**Published:** 2021-01-07

**Authors:** Andrew W. Kam, Scott Collins, Tae Park, Michael Mihail, Fiona F. Stanaway, Noni L. Lewis, Daniel Polya, Samantha Fraser-Bell, Timothy V. Roberts, James E.H. Smith

**Affiliations:** 1 Department of Ophthalmology, Royal North Shore Hospital, St Leonards, New South Wales Australia; 2 Northern Sydney Local Health District, St Leonards, New South Wales Australia; 3 Sydney Medical School, Faculty of Medicine and Health, The University of Sydney, Camperdown, New South Wales Australia; 4 Department of Ophthalmology, Westmead Hospital, Westmead, New South Wales Australia; 5 Sydney School of Public Health, Faculty of Medicine and Health, The University of Sydney, Camperdown, New South Wales Australia; 6 Department of Ophthalmology, Macquarie University Hospital, North Ryde, New South Wales Australia

**Keywords:** Ophthalmology, Service improvement, Lean, Six Sigma, Lean Six Sigma, Patient waiting times, Outpatients, Public health

## Abstract

**Background:**

Increasing patient numbers, complexity of patient management, and healthcare resource limitations have resulted in prolonged patient wait times, decreased quality of service, and decreased patient satisfaction in many outpatient services worldwide. This study investigated the impact of Lean Six Sigma, a service improvement methodology originally from manufacturing, in reducing patient wait times and increasing service capacity in a publicly-funded, tertiary referral outpatient ophthalmology clinic.

**Methods:**

This quality improvement study compared results from two five-months audits of operational data pre- and post-implementation of Lean Six Sigma. A baseline audit was conducted to determine duration and variability of patient in-clinic time and number of patients seen per clinic session. Staff interviews and a time-in-motion study were conducted to identify issues reducing clinic service efficiency. Solutions were developed to address these root causes including: clinic schedule amendments, creation of dedicated postoperative clinics, and clear documentation templates. A post-implementation audit was conducted, and the results compared with baseline audit data. Significant differences in patient in-clinic time pre- and post-solution implementation were assessed using Mann-Whitney test. Differences in variability of patient in-clinic times were assessed using Brown-Forsythe test. Differences in numbers of patients seen per clinic session were assessed using Student’s t-test.

**Results:**

During the baseline audit period, 19.4 patients were seen per 240-minute clinic session. Median patient in-clinic time was 131 minutes with an interquartile range of 133 minutes (84–217 minutes, quartile 1- quartile 3). Targeted low/negligible cost solutions were implemented to reduce in-clinic times. During the post-implementation audit period, the number of patients seen per session increased 9% to 21.1 (*p* = 0.016). There was significant reduction in duration (*p *< 0.001) and variability (*p* < 0.001) of patient in-clinic time (median 107 minutes, interquartile range 91 minutes [71–162 minutes]).

**Conclusions:**

Lean Six Sigma techniques may be used to reduce duration and variability of patient in-clinic time and increase service capacity in outpatient ophthalmology clinics without additional resource input.

## Background

Medical services worldwide face an aging population and with it, an increasing burden of disease [1]. Continuous improvement in diagnosis and management is resulting in better patient outcomes, but also increasing demands on healthcare resources. Together, increasing patient numbers, increasing complexity of patient assessment and management, and limitations on healthcare resources have resulted in prolonged patient wait times, decreased quality of service, and decreased patient satisfaction in many outpatient services across many medical specialities in both developed and developing nations [2–4]. With a focus on improving workflows, process efficiency, and reducing variability in production/service delivery, Lean and Six Sigma are two well-known management methodologies from manufacturing that may be used to help address these growing issues in outpatient healthcare settings [5, 6].

Lean, derived from the Toyota Production System, is a process improvement methodology focused on reducing ‘waste’ (steps that do not add value to the final service/product) to improve efficiency. ‘Waste’ is typically considered in 7 categories being: waiting, unnecessary transport, unnecessary human motion, inventory, over-processing, rework, and overproduction [5]. Examples of ‘waste’ in outpatient clinics include patients waiting (inventory), inappropriate testing (overproduction), or idle staff (waiting). As the patient journey through an outpatient clinic is similar to a production process, with creation of relative value units through multiple steps e.g. patient check-in, initial nursing/allied health evaluation, ophthalmologist examination, and check-out, Lean techniques may be adapted to optimise patient flow and reduce ‘waste’ [5].

Six Sigma, originally developed by Motorola in 1986, is a structured methodology to identify and eliminate defects, and reduce variation in production processes. The methodology consists of five steps [5]. Define, where issues in a process are defined from business and customer perspectives; Measure, where the process is broken down and explored; Analyse, where data is analysed to identify underlying root causes of issues; Improve, where solutions are developed, piloted and implemented to address root causes; and Control, where solutions are sustained through process control plans and ongoing monitoring. Outpatient clinics often have a high degree of variability contributing to clinic inefficiency e.g. different pathologies, differing clinician preferences etc. Six Sigma focusses on minimising variability where possible to streamline processes.

Due to their overlap, Lean and Six Sigma are often combined in a “Lean Six Sigma” approach. In recent times, Lean Six Sigma has been increasingly applied in healthcare [7]. There are few studies, however, examining its efficacy in improving publicly-funded, outpatient ophthalmology services [5, 8]. This project studied the effect of applying Lean Six Sigma in a publicly-funded tertiary referral outpatient ophthalmology service to reduce duration and variability of patient in-clinic times and improve service efficiency.

## Methods

### Practice setting

Royal North Shore Hospital Eye Clinic is a publicly-funded multi-subspecialty outpatient ophthalmology service in Sydney, Australia. Over 8,000 appointments are seen every year across 6 subspecialties, with referrals received from primary care and specialist doctors, optometrists and general ophthalmologists. The clinic also provides ‘on-call’ ophthalmic care to inpatients of Royal North Shore Hospital (> 600 beds) and patients presenting to emergency departments across the Northern Sydney Local Health District (> 185,000 presentations/year).

The clinic runs nine half-day sessions (240 minutes) every week. It is staffed by a roster of eight consultant subspecialist ophthalmologists (one on the floor for each subspecialist session and one always ‘on-call’), three ophthalmology registrars (two for all sessions, one of which is ‘on-call’ for emergency and inpatient consults), six nurses (two for all sessions) and one orthoptist (for all sessions). In any session, patients are evaluated in a multi-step process including check-in, screening (nursing/orthoptic staff assessment), investigations, ophthalmologist review and check-out. Between each step, if patients are not passed directly onto the next staff member immediately, they are returned to the waiting area or sat outside the next applicable room in the patient journey (e.g. outside the investigation room or the ophthalmologist’s room).

Within the clinic there are three rooms for screening, three rooms for ophthalmologist review, two rooms for investigations and two rooms for procedures. When a session is in progress, all rooms are dedicated to that session alone. In general, patients are booked into planned appointment slots within a session. When emergency or inpatient consults are requested however, they may be fit in on an ad hoc basis depending on clinical urgency.

### Key measures (“Define” phase)

This study’s outcome measures were: duration (median) and variability (interquartile range) of patient in-clinic time, and number of patients seen per session pre- and post-implementation. Patient in-clinic time was defined as the number of minutes from whichever was later of the appointment time, or the patient check-in time, until patient check-out. This was done to reduce the effect that patients arriving early (in which case appointment time was used) or late (in which case check-in time was used) to their appointments had on variability of in-clinic time.

### Data collection

Cerner Scheduling Appointment Book (Cerner, North Kansas City, USA), was used to schedule patient appointments. This program allowed creation of a timetable with specific appointment times and types (e.g. new, follow-up, emergency etc.) for patients to be booked into. When patients attended appointments, it recorded the time patients were checked-in and checked-out by administrative staff. Waiting time before check-in or after check-out (e.g. waiting for transport) was not captured.

Two five-month data audits of all attended appointments were conducted to determine the efficacy of the Lean Six Sigma process. A baseline audit (“Measure” and “Analyse” phases) was retrospectively conducted from February 1st to June 30th 2018. A post-implementation audit (“Control” phase) was conducted from February 1st to June 30th 2019.

### Data analysis

Patient age, gender, appointment time, appointment type, check-in time and check-out time were captured. Appointments with incomplete time data or coding errors (i.e. visits with no end time or total duration of 0 or greater than 480 minutes) were included in the count of patients seen but excluded from analysis of duration and variability of patient in-clinic time.

Difference in duration of patient in-clinic times pre- and post-implementation was assessed using Mann-Whitney-U test on SPSS (v24, IBM Corporation, Armonk, USA). Difference in variability of patient in-clinic times was assessed using Brown-Forsythe test on Excel (Microsoft, Redmond, USA) [9]. Difference in number of patients seen per session was assessed using Student’s test (SPSS). Differences in the proportions of patient appointment types seen were assessed using chi-squared tests, with Z-tests (with Bonferonni correction) used to compare pairwise differences between pre- and post-implementation proportions of appointment type (Excel). Difference in mean ages of patients with valid versus invalid in-clinic time data was assessed using Student’s t-test while differences in proportions of gender were assessed using chi-squared test (SPSS).

### Process flow maps and time-motion analysis

**Fig. 1:**
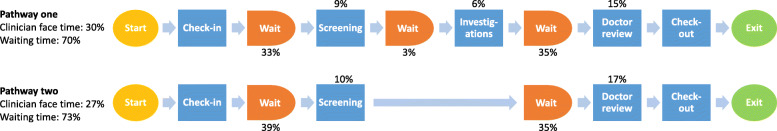
Patient flow through the Eye Clinic and the associated proportion of time spent. In both pathway one and two, over 70% of patient in-clinic time was spent waiting. Note: numbers do not sum to 100% due to rounding

Two patient process flows fit most patient journeys through the clinic; one where investigations were performed, and one without investigations. Process flow maps outlining steps in these journeys were created (Fig. 1).

A two-week time-in-motion study was conducted from June 11th to June 24th 2018 to determine proportions of total in-clinic time spent in each step along the patient journey. In this time-in-motion study, staff members noted the times they commenced and ended their roles in the patient journey on a dedicated audit document. Time between each staff member’s contact time was treated as waiting time.

The time-in-motion study data was analysed in Excel. Visits with coding errors (i.e. no time entered, times with inconsistent patient flow) were excluded. Proportions of total in-clinic time were determined and superimposed on patient process flow maps to identify bottlenecks in the patient journey (Fig. 1).

### Root cause analysis

Staff interviews, workshops, and review of patient complaint data were used to identify issues causing prolonged duration and increased variability of patient in-clinic time and clinic inefficiency. Following this, root cause analysis of issues was undertaken using the “Five Whys Technique” [10]. Resulting root causes were grouped and the most common root causes targeted for solution development.

## Results

### Baseline audit (“Measure” and “Analyse” phases)

During the baseline audit period there were 3624 visits over 187 240-minute sessions (average 19.3 patients/session). Of these visits, 2241 had valid time data for analysis. Median patient in-clinic time was 131 minutes and the interquartile range 133 minutes (84–217, quartile 1- quartile 3). Of visits with invalid data, 13 had invalid in-clinic times (due to patients arriving, being seen and discharged before their appointment time), while the remaining 1370 had invalid check-out times (checked-out the following day). Comparing invalid to valid data cohorts, there were no significant differences in age (invalid: 58.4 ± 23.2 years; valid 58.0 ± 23.4 years, *p* = 0.568) or gender (invalid: female 49.2%; valid: female 48.8%, *p* = 0.743), and only minimal differences in proportions of appointment types (Table 1).

**Table 1:** Pre vs. post-implementation appointment types

	Pre-implementation (all visits) [n, %]	Pre-implementation (visits with valid data only) [n,%]	Pre-implementation pairwise p (all visits vs. valid only)	Patient in-clinic time (minutes) [median, Q1-Q3]	Post-implementation (all visits) [n,%]	Post-implementation (visits with valid data only) [n, %]	Post-implementation pairwise p (all visits vs. valid only)	Patient in-clinic time (minutes) [median, Q1-Q3]	Pairwise p (pre-implementation vs. post-implementation total cohort)
Cataract New	107 (3.0%)	60 (2.7%)	0.442	188, 121–269	129 (3.3%)	120 (3.4%)	0.767	125, 84–179	0.186
Emergency referral	368 (10.2%)	214 (9.5%)	0.343	119, 79–184	442 (11.5%)	378 (10.8%)	0.235	109, 70–159	0.013
Follow-up	2455 (67.7%)	1555 (69.4%)	0.096	132, 89–210	2442 (63.4%)	2249 (64.4%)	0.193	105, 71–157	*<0.001
Inpatient referral	245 (6.8%)	91 (4.1%)	*<0.001	213, 123–311	308 (8.0%)	250 (7.2%)	0.070	117, 67–220	*0.006
New	248 (6.8%)	144 (6.4%)	0.434	161, 106–252	290 (7.5%)	263 (7.5%)	0.984	132, 102–179	0.119
Post-op day one	201 (5.5%)	177 (7.9%)	*<0.001	75, 49–146	242 (6.3%)	230 (6.6%)	0.451	78, 64–104	0.068
Total	3624	2241		131, 84–217	3853	3490		107, 71–162	

For all pairwise comparisons *p* significant at < 0.008 (Bonferroni correction); * significant post-hoc test

In the pre-implementation audit period, there were some slight differences in proportions of types of patients seen between the valid data cohort and the total cohort. Comparing appointment types of all patients seen pre- and post- implementation, there were some minor differences in proportions of follow-up patients seen due to restructuring of the clinic timetable

There were 329 visits during the two-week time-in-motion study. Of these, 195 had valid data for analysis. Two bottlenecks within the clinic were identified. The first, between patient check-in and screening, accounted for 33–39% of total in-clinic time depending on the care pathway. The second, before seeing the ophthalmologist, accounted for 35% of total in-clinic time. Overall, over 70% of patient in-clinic time was spent waiting in both care pathways (Fig. 1).

**Fig. 2:**
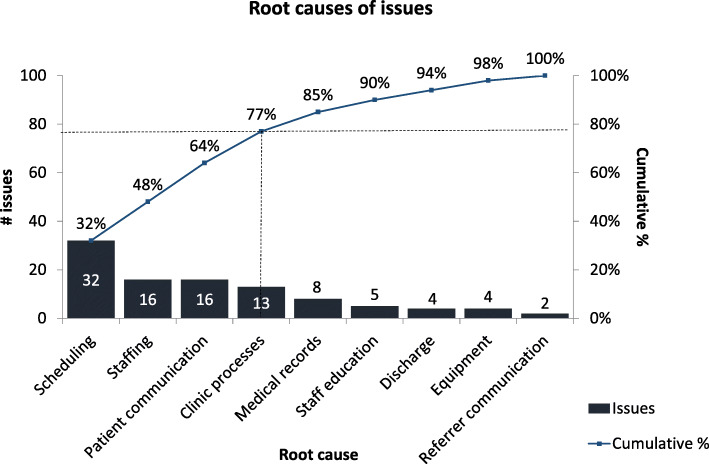
Root causes of issues in the Eye Clinic. Of issues encountered in the clinic, 77% were due to 4 root causes: scheduling, staffing, patient communication and inefficient clinic processes. These root causes were targeted in solution development and implementation

Through ten patient interviews, ten staff interviews, two staff workshops (including all staff working in the clinic), and an audit of patient complaint data, 100 unique issues causing prolonged patient in-clinic time and clinic inefficiencies were identified. Ten common root causes emerged from root cause analysis, with four contributing to 77% of issues encountered (Fig. 2).

**Fig. 3:**
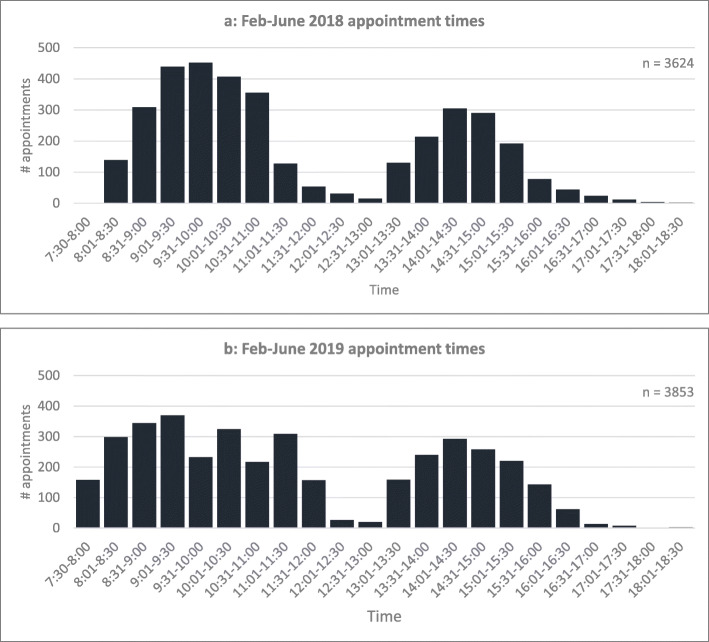
Appointment times February-June 2018/2019.Note: morning clinic sessions ran from 8am to 12 pm, afternoon clinic sessions ran from 12:30 pm to 4:30 pm. Prior to solution implementation (Fig. 3**a**), most patients were scheduled to arrive in the middle of clinics (between 9:00am-10:30am for the morning clinic and 2:00 pm-3:00 pm for the afternoon clinic). After solution implementation (Fig. 3**b**), patient arrival times were smoothed throughout the day

Scheduling was the most commonly occurring root cause identified in root cause analysis (32% of identified issues). Therefore, further exploration of scheduling data was undertaken. As seen in Fig. 3a, most patients were scheduled to arrive in the middle of clinics. This was due to the clinic schedule design, and ad hoc addition of inpatient and emergency patients into already fully-booked sessions through the clinic’s ‘on-call’ service. Patient influxes at these times were the primary contributor to the bottleneck at the start of the care pathway between check-in and screening.

### Process improvements (“Improve” phase)

Four main root causes: scheduling, staffing, patient communication, and clinic processes, were responsible for 77% of issues encountered (Fig. 2). Although funding was not available to address staffing, several other targeted negligible cost interventions were implemented to address the remaining three main root causes.

To address poor patient scheduling, the clinic schedule was revised to control patients’ arrival times. This involved: moving the start time of screening staff and patient appointments to 7:30am so patients could be screened and ready to see the ophthalmologist at 8am; revising appointment slot time lengths to better align with the needs of each appointment type; creating dedicated ‘on call’ emergency and inpatient appointment placeholders to reduce ad hoc scheduling of these patients; and providing the ‘on-call’ registrar with a ‘live’ scheduling app to allow easier identification of available appointment slots for ad hoc bookings. Furthermore, a dedicated postoperative clinic was introduced for 1-week and 4-week postoperative follow up visits as these had low variation care pathways amenable to optimisation through grouping into a dedicated clinic. The impact of these solutions is shown in Fig. 3b.

To address inefficient clinical processes, further staff feedback was sought on potential solutions and the following three solutions developed:

#### Medications

Initially, many frequently used medications (e.g. valacyclovir, timolol, brinzolamide, preservative free lubricants) were often not readily available in-clinic. This disrupted patient flow, requiring clinicians to call the hospital pharmacy to request the medications and patients to wait for them to be delivered. To address this, imprest medication lists were reviewed and updated to include these medications. Daily checks were implemented to ensure that adequate supplies of medications were available in-clinic.

#### Triage

Initially, there was no standard order to see patients in after check-in, with different staff using different approaches. There was no prioritisation system for patients with higher clinical need, e.g. inpatients, unwell persons, and no clear instruction for paper files of newly checked-in patients to be put in appointment order in the clinic’s ‘patients to be seen’ box. As clinicians generally picked up patient files from the top of the box, patients were therefore seen out of chronological order, disrupting patient flow and increasing variability in in-clinic time. To address these issues, defined escalation criteria were made for patients with clinical or other special requirements. Clear instructions were made to put paper files of newly checked-in patients in appointment order in the ‘patients to be seen’ box. Clinicians were instructed to see all patients in order of appointment time, unless there was an urgent clinical need.

#### Investigations

Initially, there was no process to clearly document investigations needed for follow up patients at their next appointment. This resulted in inefficiency as some patients occasionally needed to return to the investigation room after seeing the ophthalmologist for further tests, whilst others underwent unnecessary non-invasive investigations. To address this, a standard clinic documentation template was introduced for investigations required at the next follow up visit. This was done with the aim of prompting clinicians to consider and order appropriate investigations in advance (Online supplement: Documentation Template).

Based on the root cause analysis finding that poor patient communication accounted for 16% of issues in the clinic, all written patient communications were reviewed. Referral acknowledgement letters were updated to provide more accurate information regarding wait times for an initial appointment. Clinic information sheets and posters were developed to inform patients what to expect during their clinic visit. Fact sheets for common ophthalmological conditions and surgical procedures were introduced to improve and standardise patient education, while also potentially reducing the clinician face time needed to provide this education. Consumer representatives were used to review and provide feedback on all revised patient communications.

### Follow up analysis (“Control” phase)

**Fig. 4:**
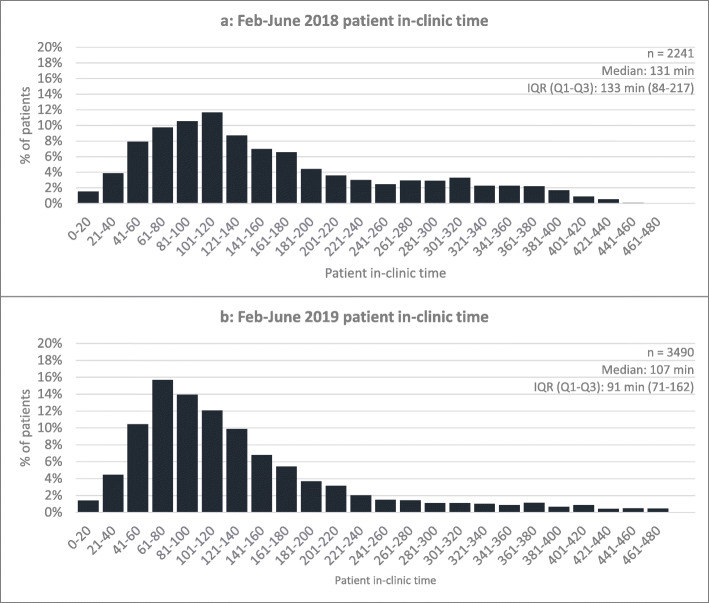
Distribution of patient in-clinic time pre- and post-implementation. Comparing pre- (Fig. 4**a**) to post-implementation (Fig. 4**b**), patient in-clinic time significantly decreased as shown through a left-shift in the distribution of in-clinic time post-implementation. IQR: interquartile range

During the post-implementation period there were 3853 clinic visits over 183 240-minute sessions (average 21.1 patients per session), a 9% increase in patients per session compared to the baseline period (*p* < 0.016). Of these visits, 3490 had valid data for analysis. Median patient in-clinic time was 107 minutes and the interquartile range 91 minutes (71–162, quartile 1- quartile 3). This was a significant reduction in duration and variability of patient in-clinic time compared to baseline (both *p* < 0.001). (Fig. 4). Of visits with invalid data, 11 cases had no check-out time, 71 cases had invalid waiting times (patients arriving, being seen and discharged before their appointment time), and 281 had invalid check-out times (checked-out the following day). Comparing the invalid to valid data cohort, there were no significant differences in age (invalid: 56.5 ± 23.3 years; valid 58.1 ± 22.8 years, *p* = 0.190), gender (invalid: female 46.5%; valid: female 43.8%,* p* = 0.321) or proportion of appointment types (Table 1).

## Discussion

In this study, application of Lean Six Sigma techniques in a publicly-funded tertiary outpatient ophthalmology clinic led to development of solutions that significantly reduced duration and variability of patient in-clinic time. Median patient in-clinic time was reduced by 18% and the interquartile range by 32%. These results were achieved while patients seen per session increased 9%. Solutions used to achieve these results were: clinic schedule amendments to prevent sudden influxes of patients, a dedicated weekly postoperative patient clinic for one week and four week postoperative visits, checks to ensure frequently used medications were always available in the clinic, defining a standard order to see patients in, clear follow-up patient investigation planning documentation templates, and patient information pamphlets for common ophthalmic conditions/surgeries. Of note, these solutions were implemented without additional capital requirements (e.g. purchasing new devices) or ongoing staffing costs.

This study adds to the growing body of literature demonstrating that techniques from business and industry, such as Lean Six Sigma, can be used in healthcare settings to improve system efficiency. Specific to ophthalmology, one North American group who applied Lean Six Sigma techniques to a subspecialist retina clinic (subsequently hiring an extra technician, creating a dedicated intravitreal injection patient pathway, and improving clinic scheduling), reduced mean patient visit times by 18% (*p* < 0.05) and variation in visit time by 5% [5]. A second North American group who applied Lean thinking (decentralising their optical coherence tomography machines from a central photography suite into technicians’ screening rooms), reduced patient wait times by 74% (*p* < 0.0001) and in-clinic time by 36% (*p* < 0.0001) [11].

Outside of ophthalmology, Lean Six Sigma has been shown to be effective in a range of healthcare contexts. The Cleveland Clinic Cardiac Catheterisation Laboratory, as an example, applied Lean Six Sigma techniques subsequently improving patient turnover times, the number of on-time patient and physician arrival times and reducing physician down times [12]. A further example was seen in Indiana pertaining to orthopaedic inpatient care at the Richard L. Roudebush Veterans Affairs Medical Centre in Indianapolis. Their group used Lean Six Sigma techniques to reduce length of stay of joint replacement patients by 36% from 5.3 days to 3.4 days (*p* < 0.001) [13]. Finally on a hospital-wide basis the University Hospital “Federico II” of Naples, used Lean Six Sigma techniques to reduce healthcare-associated infections in inpatients across multiple medical specialties including general medicine, pulmonology, oncology, nephrology, cardiology, neurology, gastroenterology, endocrinology and rheumatology [14].

Process improvement methodologies such as Lean Six Sigma, present a significant opportunity to deliver better value in healthcare through improved efficiency and reduced ‘waste’. More broadly, as demands on healthcare services continue to grow across most medical specialties, a focus on service improvement will be needed to best utilise the limited resources available. This is particularly true within publicly-funded healthcare systems where long waiting times for non-emergency services are an increasingly common feature [15].

Service improvement, particularly in organisations utilising Lean Six Sigma methodology must incorporate the feedback of all their people including patients and the multidisciplinary healthcare team. Input from the entire team not only allows for better issue identification and solution generation, but also has the potential to increase team cohesiveness and motivation to actively participate in service improvement [16]. In this study, broad staff engagement through interviews and workshops allowed a comprehensive diagnosis of issues facing the Eye Clinic, identification of suitable, low/negligible cost solutions, and motivated all staff, from check-in desk to ophthalmologists to contribute to the service improvement effort. Going forward, we believe it has helped facilitate the development of a continuous improvement culture not only in the Eye Clinic, but also more broadly in our organisation, with the lessons learnt in this study now being applied to other outpatient clinics at our hospital.

This study has several limitations. Firstly, only qualitative data (i.e. staff interviews) was used to determine inefficient clinic processes. A quantitative investigation defining exact contributions of these issues to pre- and post-implementation in-clinic times would have better clarified the efficacy of each solution. Secondly, this study did not formally measure the effect of our solutions on patient and staff satisfaction. Staff interviews suggest however, staff satisfaction and engagement in improving clinic efficiency has improved. Other studies in outpatient clinics have demonstrated that reduced patient wait times improve patient satisfaction [3]. Thirdly, as the baseline audit was performed retrospectively, many patient visits had invalid data and were excluded from in-clinic time analysis (1383 of 3624 visits). This was noted in the improvement process and the check-out process was subsequently standardised, resulting in less invalid data in the post-implementation audit (363 of 3853 visits). Overall, most invalid data was due to administration staff oversight in checking-out patients at the end of their appointment (these patients were checked-out the following day). As such, it is likely the invalid data is missing completely at random, as opposed to being missing due to patient or in-clinic time related factors. This is supported by there being no differences in age or sex between invalid and valid data cohorts, and only minimal differences in the proportion of appointment types between the total and valid data cohorts.

There are, however, many strengths to this study. Firstly, this study was conducted in a large publicly-funded tertiary referral outpatient ophthalmology service with both inpatient and emergency services, a setting at high risk of facing resource constraints. The fact that the improvements seen in this study were delivered without significant additional capital or ongoing staffing costs increases its applicability to other services with similar characteristics. Secondly, this study had a large sample size, including all patients seen, across a range of subspecialties over the audited periods. This further increases applicability of this study’s results to other large, multi-subspecialty ophthalmology services. Thirdly, as many of the solutions implemented are not specific to ophthalmology, they could potentially be applicable to other outpatient specialties. Finally, by auditing patient wait times over two corresponding five-month periods in the year (February to June), the potential for holiday periods and seasonality confounding the results was reduced.

## Conclusions

In summary, this study demonstrates that applying Lean Six Sigma to publicly-funded outpatient ophthalmology clinics can reduce duration and variability of patient in-clinic time and increase service capacity, without significant upfront capital expenditure or ongoing resource requirements. It outlines an approach to applying Lean Six Sigma that may be used in other healthcare contexts and some potential solutions that may be applicable to all outpatient clinics, ophthalmology or otherwise. As demands on healthcare resources continue to increase in the future, Lean Six Sigma techniques may play an increasingly important role in improving the delivery of healthcare services.

## Data Availability

The datasets used and/or analysed during the current study are available from the corresponding author on reasonable request.

## Abbreviations

IQR: Interquartile range
